# Supplementary material for: Are carbon emissions trading and green financial instruments synergistic? -Comprehensive quantitative research based on content analysis
Source: PLoS One. 2024 Mar 7;19(3):e0298601. doi: 10.1371/journal.pone.0298601 (PMC10919601; doi:10.1371/journal.pone.0298601)
Supplement: S1 Data — (ZIP) [file pone.0298601.s001.zip › Data/地级市Z_F工作报告.zip!/╜¡╬≈/╥╦┤║╩╨/└┤╫╘╣1⁄2╓┌║┼í╛┬φ┐╦╩2╛▌═°í┐_2003.docx]

各位代表：

现在，我代表市人民政府向大会作政府工作报告，请予审议，并请市政协各位委员和其他列席会议的同志提出意见。

一、2002年政府工作回顾

2002年，在市委的正确领导下，全市上下紧紧围绕建设赣西经济中心城市的总体目标，突出发展第一要务，实事求是，奋发努力，经济结构在困难中得到调整，财政收入在“阵痛中”实现增长，经济建设和社会事业呈现好的发展态势。完成国内生产总值221亿元，增长10.2%；财政总收入17.88亿元，增长5.7%；农民人均纯收入2582元，增加80元；城镇居民人均可支配收入6245元，增加848元；全社会固定资产投资61.5亿元，增长19%；社会消费品零售总额105.67亿元，增长8.3%。

经济发展势头良好，产业结构逐步优化。工业生产摆脱多年低速徘徊，实现快速增长，规模以上工业完成增加值24.8亿元，增长23.8%，高于全省平均水平7.8个百分点，其中丰电公司、四特集团、三九宜工等一批大中型骨干企业发挥了重要支撑作用。11个工业园区完成基础设施投入8.4亿元，入园企业283家，建成投产项目108个，上交税金7859万元，安排就业2万多人。以产权制度改革为核心的企业改革向纵深推进，多数县市国有企业改革基本完成，市直企业改革取得实质性进展，宜春造纸厂、江西机床电器厂依法实施破产，实现成功转让。农业10大主导产业实现综合产值20亿元，增长22%；实现税收5500万元，增长19%。农业产业化龙头企业在成长壮大，青龙高科、霍曼内特、宜丰猕猴桃酒业等一批龙头企业辐射带动作用日益显现。农业科技示范园初见成效，引进果蔬、水产等品种上百个。农产品基地建设发展较快，省级无公害农产品基地达到19个。农业结构调整力度加大，完成退耕还林31万亩，中药材、花卉苗木、蔬菜和特种养殖成为农民增收的新渠道。农业规模经营不断扩大，土地、山林、水面使用权流转面积240万亩。农村服务网络不断完善。个体私营经济发展取得新成效，上交税金 4.83亿元，增长15.5%，占财政总收入的27.1%，济民可信、海尔思、仁和集团、丰源公司等一批私营企业纳税超百万元，成为经济发展的重要增长点，个私经济发展工作受到省政府表彰。经济结构战略性调整取得历史性突破，三次产业比重为32.9:34.4:32.7，二产首次超过一产，三产逼近一产。

对外开放步伐加快，资金投入势头强劲。广泛深入开展了塑造江西人新形象和学习浙江经验等一系列活动；在上年的基础上又取消行政审批事项64项，总计取消率达70.8%；组建了“市经济发展服务中心”，经济发展环境进一步优化。招商引资持续升温，组织和参加了香港、温州、厦门、深圳、东莞招商推介会，组建了驻东莞、温州、泉州、萧山等地4支市直定点招商小分队，在香港、东莞、厦门的企业界和工商界聘请了4位经济协作顾问，全年实际引进境内市外资金48.3亿元，增长86.2%；实际引进境外资金4505万美元，增长70%；出口创汇2185万美元，增长9%。国内招商引资工作综合考评列全省第三名。积极开展了“争资金、争项目、争政策”活动，进一步密切了与国家和省有关部门的联系，共向上争取各类资金13亿元。着力营造“双赢”的银企关系，金融机构各项贷款余额净增6.86亿元，同比多增2.98亿元。

基础设施建设明显加快，城乡面貌不断改观。公路建设力度加大，新建、改建县乡公路231公里，万载至铜鼓、大城至干洲公路，沪瑞高速公路昌傅至金鱼石段全面开工。农村电网改造基本完成，总投入8715万元，农村生活用电价格降到每度1元以下。水利基础设施建设完成投入1.28亿元，赣抚大堤、泉港分洪闸、店下水库等重点水利工程的除险加固和潦河灌区的配套改造进展顺利，宜春、宜丰、奉新等城市防洪工程正在抓紧建设。宜春中心城争创“三城”工作正式启动，16项城建重点工程有11项完成或接近完成，完成投资5亿元。袁山公园和秀水公园相继建成开放，朝阳路改造接近尾声，宜春火车站形象得到改观，城区人行道板改造总里程达20公里，新增道路绿化面积5万平方米，城市绿化覆盖率达到37.29%。城区“三车整顿”和“三清理”工作成效明显。经营城市的理念逐步形成，公开、公正、公平招标拍卖和挂牌出让土地工作初见成效，市本级土地收益增长166%。以县城为重点的城镇建设取得可喜成绩，一批小城镇功能得到提升、配套设施得到改善、生态环境得到保护。

农运会筹办进展顺利，社会事业蓬勃发展。农运会筹办工作取得实质性进展，根据总体工作方案抓紧了分项工作的实施，确定了比赛项目和大型文体表演创意方案，一批比赛场馆和接待设施的建设及改造开始启动，筹资工作取得成效，共筹集资金857万元。在深圳成功举办了农运会新闻发布会。科技工作成效明显，争取省级以上科技项目57项，4项科研成果列入国家“863”计划和星火计划。教育事业成效突出，“两基”工作进一步巩固提高，高中阶段教育得到健康发展，作为全省唯一的基础教育先进市曾由省政府呈报国家表彰；宜春学院提前2年实现在校生规模达万人。文化工作取得好成绩，一批体现地方特色、反映宜春发展的优秀文艺作品获国家级金奖。体育工作成绩显著，在省第十一届运动会上荣获团体总分第二名、金牌总数第三名，宜春本土培养的运动员杨文军、罗贤国分别获亚运会和世青赛冠军。人口和计划生育工作明显加强，人口自然增长率控制在8.06‰。卫生工作成效明显，医疗卫生水平继续提高，农村卫生服务网络不断完善。广播电视光缆建设步伐加快，有线电视网络进一步向村一级延伸。精神文明建设扎实推进，开展了以“争做文明市民、共建美好宜春”为主题的素质教育活动。

安全生产形势明显好转，社会稳定得到有效维护。继续深化煤矿、花炮、非煤矿山、道路交通和消防安全专项整治，组建了安全监察队伍，强化了安全监管，重特大生产事故大幅减少，工矿企业发生事故和死亡人数分别下降10.2%和6.5%。深入开展了“严打”整治斗争，加强了对“法轮功”等邪教组织和敌对分子的防范和打击，社会治安形势明显好转。在一系列改革推进的过程中，没有出现重大群体事件。

社会保障体系不断完善，人民生活水平进一步提高。离退休人员养老金和下岗职工基本生活费做到了按时足额发放，城镇居民最低生活保障人数达11万多人；就业和再就业工作力度加大，城镇登记失业率控制在3.7%以内，安排下岗、失业人员再就业2.4万多人。城乡居民收入增加，储蓄存款余额达179亿元，人均储蓄存款余额增加474元。城乡市场比较繁荣，连锁店、专卖店、大型超市相继涌现。居民生活条件进一步改善，城区人均住房面积达15.72平方米；消费层次不断提高，文化、教育、旅游等正成为新的消费热点，移动电话、互联网络用户发展迅猛，分别达到48万户和9.95万户，增长34.1%和65.8%。

市级机构改革顺利完成，精简行政编制290名，县乡机构改革取得阶段性成果。农村税费改革稳妥推进，农民总体减负达32.6%，其它各项配套改革正在实施。信访接待、纠纷调解等工作得到加强。政府法制工作进一步加强，行政执法监督引向深入，依法行政水平进一步提高。政府坚持向人大报告工作，加强了同政协的联系，认真接受人大法律监督和政协民主监督；人大代表建议、政协委员提案共157件全部办复。国家安全、人民防空、国防动员、民族宗教、侨务外事、档案、史志、老龄和妇女儿童等工作都有新的进步。

这些成绩的取得，与市人大和市政协的有效监督及社会各界大力支持密不可分，是全市上下共同努力的结果。在此，我代表市人民政府，向全市各条战线的广大工人、农民、知识分子、干部，向人民解放军和武警驻宜部队官兵、公安干警，向关心和支持宜春发展的各民主党派、工商联、各人民团体和各界人士以及海内外朋友表示崇高的敬意和衷心的感谢！

各位代表，一年来的成绩，为今后的发展奠定了良好的基础，进一步坚定了我们加快发展的信心。但我们也清醒地看到，前进的道路上仍有不少困难和问题，突出表现在：经济结构仍不够合理，经济增长点还不多，政策性减收因素比较集中，财政增收有难度；农业产业化水平仍然不高，农民增收渠道不广，增收幅度不大；投资环境仍不尽人意；就业和再就业压力仍然较大，部分下岗失业人员和一些特殊困难群众生活还比较艰苦；高危行业仍大量存在，改革深层次矛盾亟待解决，安全稳定工作任务仍很繁重；县域经济发展不平衡，有的县市之间差距比较大。对这些困难和问题，我们一定高度重视，继续采取有力措施，认真加以克服和解决。

二、2003年政府工作安排

2003年是全面贯彻落实党的十六大和十届全国人大一次会议精神，开创我市各项工作新局面的重要一年，也是全面建设小康宜春的开局之年。做好今年的政府工作，具有十分重要的意义。

今年政府工作的总体要求是：以邓小平理论和“三个代表”重要思想为指导，全面贯彻党的十六大精神，紧紧围绕加快建设赣西经济中心城市和全面建设小康宜春的总目标，坚持以发展为先导，以创新为灵魂，以富民为根本，进一步深化改革、扩大开放，主攻工业发展，放活民营经济，推进城镇经营，力促农民增收，齐办全国农运，狠抓安全稳定，统筹城乡经济社会发展，促进全市经济持续快速健康发展和社会全面进步。

全市经济和社会发展的主要目标预期为：国内生产总值增长11%，财政总收入增长9%，农民人均纯收入增加100元，城镇居民人均可支配收入增加400元，全社会固定资产投资增长22%，个私经济税收增长25%，实际利用外资增长56%，利用市外资金增长41%，外贸出口增长28%，社会消费品零售总额增长10%，城镇登记失业率控制在4.5%以内，人口自然增长率控制在8.5‰以内，城镇化率达到29%。

根据上述总体要求，为了实现今年的经济和社会发展目标，我们将着重抓好以下九个方面的工作：

（一）着眼于建设小康宜春，开好头、起好步，全力优化区域经济布局

按照省委书记孟建柱同志提出的“把宜春建设成为一个产业结构比较合理，县域经济综合实力比较雄厚，生态环境比较优雅的赣西经济中心城市”的目标定位，全面建设小康宜春，关键是进一步优化生产力布局，根本是不断壮大综合实力。去年政府工作报告提出了“一个中心一个圈，两个园区一走廊”的构想，经过一年多的调研论证和实践，基本形成了“一心一圈两走廊”的发展布局构想。

“一心”：做强做大宜春中心城市，使之成为宜春经济发展最重要的增长极。做强产业基础，做大城市规模，做美人居环境。积极实施“外拓内改，三城共创”，新城建设与旧城改造同步进行，努力创建园林城、卫生城、文明城，为迎接第五届全国农运会召开创造优美的城市环境，提供完善的城市设施。依托两个工业园区和现有优势骨干企业，发展壮大工业经济，打造优势突出、特色明显的企业集群和产业簇群，夯实中心城市的产业基础，增强中心城市的聚集、辐射功能。

“一圈”：发展丰城、樟树、高安城镇经济圈，使之成为宜春经济的“金三角”。按照中等城市标准，扩大城市规模，完善城市功能，加快高等级公路和信息网络体系建设。根据各自优势和特点，搞好经济分工协作，加快支柱产业建设，做到优势互补、资源共享、相互促进、各具特色，建设成为全省最具特色、最具实力的城镇经济圈，成为江西崛起的“排头兵”。

“两走廊”：建设320国道经济走廊和山区生态经济走廊，使之成为特色明、活力足的经济增长带。建设320国道经济走廊，突出抓好沿线工业园区、农业科技示范园区和以县城为重点的城镇建设，依托优势产业培育专业批发市场，抓好沿线优质农产品、工业原料林等基地建设，形成人流、物流、资金流、信息流的快速通道。建设山区生态经济走廊，充分挖掘和发挥山区生态资源富集、矿产资源富集、佛教禅宗文化富集等优势，加快发展特色经济和旅游产业，成为宜春经济夺目的亮点。

（二）着眼于蓄积发展后劲，走出去、请进来，全力加大资金投入

经济发展千条线万条线，投入是第一保障线。要千方百计增加投入，努力保持较高的投资水平和较大的投资规模，增强投资对经济发展的拉动作用。

全力以赴招商引资。按照“顺应入世，承接沿海，远学浙江，近联两南（南昌、湖南），提升实力，加快发展”的开放格局，主动走出去，积极请进来，确保完成引进境外资金7000万美元、引进市外境内资金68亿元的目标。继续坚持政府推动、部门参与、社会联动，不断扩大招商引资的社会基础。充分发挥驻外办事处的“窗口”作用、市直招商专业小分队的“先锋”作用和市政府经济协作顾问的“桥梁”作用，使之成为推介宜春、招商引资的主力军。继续坚持和强化目标管理，完善激励机制，对完成和超额完成招商引资任务的单位和个人及时兑现奖励，功高者重奖；对没有完成任务的通报批评，并按规定予以处罚。

加快步伐跑省进京。高度重视向上争资金、争项目、争政策工作，进一步落实责任，强化措施，以抓招商引资的精神和干劲，加大跑省进京的力度，争取中央和省更多的支持。根据国家产业政策和投资导向，结合宜春实际，找准切入点，提出和储备一批项目，加大项目前期工作力度，努力争取上级资金投入。大力宣传宜春，扩大宜春影响，坚持和完善定期走访制度，与更多的中央、省属部门建立密切友好联系，为“三争”工作打下良好基础。

努力拓宽投资渠道。积极启动民间投资，放宽民间投资市场准入领域，消除民间投资的体制性障碍，多方面入手扩大民间投资，增强群众的投资创业意识。努力争取信贷资金，加大对工业园区、农业主导产业、民营经济的投入。积极协调银企关系，努力形成银企“双赢双活”的良性循环。进一步盘活资产存量，重点盘活城市土地资产、企业闲置资产，努力经营好国有无形资产和公用设施。大力开展政府信用、企业信用和个人信用建设，打造“信用宜春”。

（三）着眼于加速工业崛起，建园区、壮支柱，全力主攻工业

宜春要发展，核心是工业要发展。围绕壮大工业经济总量，提升工业经济运行质量，集中力量加快工业园区建设，培植壮大支柱产业，加大技术创新力度，改造提升传统产业，全力推进新型工业化。

加快工业园区建设和发展。紧紧抓住沿海地区产业向内地转移的机遇，创新招商方式，拓宽引资领域，力争全市工业园区企业新增200户，投资总额新增25亿元，上交税收1亿元，提供就业岗位3万个。坚持“财政启动、资本经营、多方投资、市场运作”的原则，加快园区基础设施建设，推进园区管理体制由建设型向经营型转变。加强对签约项目的跟踪服务，加快入园企业建设进度。抓好园区配套服务体系建设，对入园企业提供项目立项、用地报批、工程报建等“一站式”服务。

培植壮大工业支柱。继续抓好《关于培植壮大支柱产业加快工业经济发展的实施意见》的落实，重点扶持机械、食品、建材、竹木加工、医药等五大产业发展，提高机械行业的产品研发能力，继续抓好食品工业，积极开发新型建筑材料，大力发展农林产品多层次的精深加工，加快医药产业发展。

加大科技创新力度。引进一批先进适用技术，培育一批具有较高科技含量和自主知识产权的产品，尽快形成产业规模。利用高新技术改造传统产业，注重提高主导产业和优势产业的技术装备水平，促进整个工业装备更新和技术水平的提高。坚持质量兴企，积极实施名牌战略，努力培植名牌产品，争创著名商标。加快信息化建设与应用步伐，走新型工业化道路。

（四）着眼于推进农业产业化，扩规模、上水平，全力推进农村经济大发展

发展农村经济，增加农民收入，维护农村稳定，是全面建设小康宜春，促进整个国民经济持续快速健康发展的重大问题。必须跳出农业来发展农业，开放农村来繁荣农村，减少农民来富裕农民。

发展农业主导产业。继续抓好十大主导产业。突出抓好奶肉牛工程、林业产业和有机农产品，重点扶持霍曼内特、青龙高科、江麻和宜丰猕猴桃酒业等龙头企业。努力吸引各方面资本参与农业开发，领办、创办和参与经营龙头企业。注重打造优质农产品品牌，争取对部分名优特地方产品申请国家原产地域保护。加强宣传造势，扩大龙头企业知名度，通过龙头企业连农户、带基地，牵动产业发展。

加快农产品基地建设。大力发展以杨树和丛竹为主的速生丰产林，加快工业原料林基地建设。以建设320国道宜春段花卉苗木长廊为重点，形成3万亩左右的花卉苗木基地。大力发展中药材种植，形成25万亩的中药材基地。大力推进无公害农产品生产基地建设，争取市级以上认定的无公害生产示范基地总数达到55个。

促进农村经济可持续发展。规范农村用地和建房，加快农村沼气工程建设，抓好人畜饮水工程建设，积极组建农产品检测中心，加快我市农产品质量检测与国际接轨的步伐，抓好农业科技示范园建设，加强农经信息队伍建设。

（五）着眼于实现富民强市，激活力、创特色，全力促进民营经济大提高

民营经济是国民经济最具活力的增长点，要创造一个宽松的政策环境和良好的社会氛围，让一切创造社会财富的源泉充分涌流，使民营经济成为推动我市经济发展的重要力量、财政增收的重要来源、扩大就业的重要渠道。

提高民营经济比重。进一步放宽经营领域，降低准入门槛，拓宽发展空间，大幅度提高民营经济在国民经济中的比重。除国家法律法规明令禁止的行业和领域外，允许民营经济自主选择经营范围和经营方式。鼓励民营企业参与基础设施和公益事业项目的投资与经营，支持民营企业参与社会事业和社会服务业的建设和发展，引导一批有实力的民营企业参与农业产业化经营，吸引市外民营企业家到我市投资兴业，实行同等待遇，鼓励公平竞争。

提高民营企业科技含量。通过政策引导，重点扶持一批科技含量高、生产规模大、竞争能力强、发展前景好的民营企业。引导民营企业发展主导产品，打造优势品牌。鼓励和引导本地科技人员兼职创办民营科技企业，或从事高新技术成果的转化工作。大力构筑政策、技术、信息服务平台，将民营企业引入高新技术产业发展的新领域，引向现代企业发展的大方向。

提高民营企业经营水平。提供优厚待遇，吸引外地一批懂经营、善管理的高层次人才来宜春引领民营经济发展。加强对本地民营企业高级管理人员的培训，有针对性地组织民营企业家走出江西、走出国门，开阔视野，提高素质。贯彻落实已有的规定，并从实际出发，创造更加宽松、更加灵活的政策环境，鼓励大中专毕业生、机关事业单位干部大胆下海，兴办个私民营企业。

（六）着眼于激发内生活力，顺体制、活机制，全力推进改革新突破

发展无止境，创新无止境。做好今年的政府工作，关键要在改革上求突破，在创新上求发展，革除体制障碍，消除机制弊端，使蕴藏在各方面的潜能和创造力尽可能得到最大发挥，成为推动经济快速发展生生不息的源头活力。

加快行政管理体制改革。进一步巩固机构改革成果，继续精简行政审批事项，凡不符合市场经济要求的原则上全部取消，已经取消的坚决落实到位。按照建立公共财政体系要求，加大培植财源力度，深化财政体制改革，完善市与县、县与乡财政管理体制，改革预算编制方法，实行综合预算和零基预算，开展部门预算试点，推进国库集中收付制度改革，依法实行政府采购，深化“收支两条线”管理。坚持依法理财、依法治税，建立税收监管机制，优化财政支出结构，保障工资、社保及其他重点支出的需要。积极推进依法行政，进一步健全行政执法责任制和评议考核制度、政务公开制度、行政人员过错责任追究制度、行政复议制度。继续推进“双优”工作，下大力气改善投资软环境。加紧完善市经济发展服务中心功能，打造“政务超市”。建立健全投资与服务协调督查机制，依法严厉打击各类破坏投资环境的行为。积极稳妥地推进公路管理体制改革，促进公路建设更快发展。

深化企业改革。进一步抓好重点企业的股份制改造，完善江特公司、江西猕猴桃酒业公司的法人治理结构和内部管理机制，争取早日上市。加快市直企业改革进度，尽快完成江西机床电器厂破产扫尾，扶持新公司发展壮大。加快汽运公司、江麻公司、长青公司和宜齿公司改制步伐。进一步加快流通企业和其他非工口企业的改革进度，尽早完成改制工作。

推进农村改革。巩固和扩大税改成果，依法加强农业税及其附加的征收管理，积极稳妥地推进农村税费配套改革，切实减轻农民负担。稳定和完善土地承包关系，推进土地、山林、水利设施产权制度改革，在确保农民土地使用权的基础上，促进土地资源有序合理流转，为农业产业化经营和农业规模经营创造条件。创新农业投融资机制，探索加大农业投入的新路子，加强农业资金的监管，确保专款专用。

抓好社会事业改革。深化教育改革，巩固提高“两基”成果，引导社会力量举办非义务阶段教育；积极稳妥地实施教师聘任制改革，基本完成农村中小学布局结构调整工作。抓好科技体制改革，加强科技、教育与经济的结合，大力实施科教兴市战略。深化文化改革，抓好卫生改革，打响宜春“体育牌”，加快广播电视事业发展，抓好人口和计划生育工作，确保完成省政府下达的人口计划生育目标。

（七）着眼于充分利用两个市场，拓外贸、活内贸，全力搞活商品大流通

发展开放型经济，主动参与国际分工与竞争，积极扩大区域交流与合作，提高生产与流通的关联度，提高国民经济对外贸的依存度，努力构建大流通格局。

努力扩大外贸出口。积极推进出口主体和出口市场多元化，努力形成自营出口生产企业、外商投资企业、外贸公司“三路大军”齐上的出口创汇新格局。落实出口鼓励政策，重点扶持劳动密集型、科技含量高、农业产业化龙头出口企业，帮助他们争取国家外贸发展基金、技术改造贷款贴息资金、国际市场开拓资金支持。积极鼓励、动员并协助具备条件的各类企业申报自营进出口权。

积极搞活内贸流通。培育和发展专业批发市场和生产资料市场，重点建设和培育一批龙头批发市场，促其尽快上规模、上档次、上水平，增强市场的竞争力和辐射力。引进和发展连锁经营、物流配送和电子商务等新兴业态，抓紧培育一批组织化、规模化、现代化程度较高和竞争力较强的流通企业，建设区域性物流中心和社区商业中心。

发展民间流通组织。运用信息技术，创新经营理念，发展和扶持一批现代化农产品流通组织，重点支持和培育一批实力较强、经营者素质较高的民间流通企业，使其在搞活农产品流通中发挥作用。加快流通行业协会的培育建设，充分发挥其中介作用。

（八）着眼于抓好农运会筹办，出精品、树形象，全力打造宜春品牌

高标准筹办第五届全国农运会，是宜春经济实力的大展现，是宜春城市形象的大展示，是宜春人民精神风貌的大展阅。要举全市之力，干出精神，干得精明，干出精品，把农运会办出时代特征、办出“三农”特性、办出宜春特色，并以此为契机，从抓好城市建设与管理、基础设施和市政公用设施建设、特色旅游业发展等方面入手，彻底改变宜春形象，把宜春的品牌树起来，把宜春的声誉扬出去。

抓好农运盛会筹办。按照“声势大于绵阳，接待近于绵阳，环境好于绵阳，大型文体表演优于绵阳”的要求，高标准抓好各项筹办工作。重点抓好文明创建、场馆及宾招设施的建设改造、大型文体表演创作和排练、对外宣传、资金筹措、赛事组织等工作。完成市游泳馆、宜春学院体育场馆建设，以及体育中心、水上运动基地等比赛场地的维修改造，新建和改造一批接待设施，组织开幕式文体表演排练，完成闭幕式文艺晚会的创作，组织好比赛项目的训练以及裁判员培训，适时在南昌、北京等地举行新闻发布会。

改善宜春城市形象。努力推进城市化进程。按照“城乡资本重组，提高资本效率；实行市场化运作，提高经营水平；打造个性特色，提高城市品位”的要求，加快以县城为重点的城市建设步伐，提高城市化水平。突出抓好宜春中心城市建设与管理。加快迎宾大道、新行政中心和新区道路建设，拓展城区面积；大力实施“整装美容”、“设施配套”、“青山秀水”、“蓝天白云”、“文化再现”工程，加快老城改造。启动城市管理综合执法，推进城市公用事业市场化运作和产业化发展。改革城镇户籍管理制度，降低农民进城门槛，引导农民及外来人员到城镇发展创业，确保年内城镇人口增加10万人，努力聚旺城市人气，促进城市繁荣和发展。加大各县市城区及重点集镇的建设与管理力度，提高管理水平，力争年内城镇面貌有看得见的变化。继续深入开展精神文明创建活动，推动市民素质和城市文明程度的提高。

加快基础设施建设。抓紧完善“三纵三横”公路交通主骨架，新建、改新中国成立、省道及县乡公路766公里，重点抓好老区公路、国债公路和旅游景区公路建设。加快躁周线、上铁线、干大线、修铜线、芳万线建设，力争完成105国道丰城、樟树境内的改线工程，抓紧320国道宜万段、袁州区沙温线、奉新靖安万黄线、高安建新线的开工建设。积极配合赣粤高速公路昌傅段、沪瑞高速公路宜春境内段建设。着手开展第三期农网改造，突出抓好县城电网建设与改造。抓紧赣抚大堤加固工程收尾、锦惠渠灌区、潦河灌区及病险水库除险加固等一批水利工程的建设。实施袁河水厂一期工程、宜春污水处理厂一期工程、外环南路等重点工程，推进人防设施建设，促进宜春中心城市功能的完善。

推进旅游产业发展。全面实施《宜春市旅游业发展总体规划》，加紧编制各景区控制性详规，多元化、多渠道筹集资金，加快景区的开发建设，重点抓好明月山、三爪仑、天柱峰以及阁皂山等景区建设，提高旅游景区建设的品位和档次。积极组织申报省级风景名胜区，认真抓好景区景点质量等级评定，精心培育一批旅游示范点。壮大从业队伍，加强宣传促销，进一步拓宽客源市场，增加旅游收入，提高宜春旅游知名度。

（九）着眼于保障改革发展，同心抓、协力管，全力抓好安全维护稳定

正确处理改革发展稳定的关系，是一项十分重要的系统工程。政府各部门都应同心协力，齐抓共管，进一步加强安全监管，做好就业和再就业工作，切实维护社会稳定，为改革和发展创造良好的社会环境。

加强监管，确保安全。高度重视安全工作，保持高度警惕，形成高压态势，努力实现“两杜绝，一减少”目标。继续深化煤矿、花炮、非煤矿山、道路交通和消防安全等重点行业的专项整治。发挥市县两级安监队伍的作用，加强督查，依法查处违章行为。进一步落实安全监察员制度和县、乡两级干部挂点负责制，确保安全生产各项制度和措施落到实处。

抓好综合治理，全力维护社会稳定。继续按照中央和省部署，依法打击极少数“法轮功”等邪教组织违法犯罪活动，确保实现“进京滋事”、在当地聚众闹事和插播电视三个“零指标”。抓好社会治安综合治理，加大“严打”整治斗争力度，强化社会面控制。进一步健全快速高效处置突发性重大事故、群体事件的工作机制。继续整顿和规范市场经济秩序，打击制售假冒伪劣商品的违法犯罪行为。严厉查处公路“三乱”。广泛开展法制和道德教育，增强企业和公民的法制观念和商业道德观念。做好信访工作，妥善处理人民内部矛盾。

扩大劳动就业，完善社会保障。积极组织开展大型招聘活动，重点做好有劳动能力和就业愿望的下岗失业人员尤其是“4050”人员的再就业工作。注重引进发展劳动密集型、服务型企业，社区保洁、保绿、保安、保养等公益性岗位优先安排下岗失业人员，确保为5万人提供就业岗位。抓好专业技能培训，力争培训下岗失业人员3万人。扶持建立再就业基地30个。建设一批有一定规模和较高标准的劳动力市场，继续打造“宜春民工”品牌，扩大省外劳务输出。落实“两个确保”，抓好“一个低保”，关心困难群众特别是弱势群体的生产和生活。

加快建设赣西经济中心城市，全面建设小康宜春，我们深感责任重大，任务艰巨。政府各部门和工作人员都必须紧紧抓住“第一要务”，时刻牢记“两个务必”，身体力行“三个代表”，解放思想，提高素质，履行职责，扎实工作，不负重托，不辱使命，做到权为民所用、情为民所系、利为民所谋。顺应时代要求，加强学习，提高驾驭经济全局的能力，提高科学决策水平，建设学习型政府。加强政府法制建设，增强法制观念，坚持依法行政，自觉接受市人大及其常委会的依法监督、人民政协的民主监督和社会各界的监督，加强与各民主党派的联系，建设法制型政府。转变作风，求真务实，畅通与群众沟通联系的渠道，大力推行政务公开，提高工作效率，建设服务型政府。坚持反腐倡廉，加强廉政建设，规范政府工作人员特别是领导干部的行政行为，从严治政，建设廉洁型政府。

各位代表，2003年是充满希望和挑战的一年，新的形势催人奋进，新的事业任重道远。让我们高举邓小平理论伟大旗帜，沿着党的十六大指引的方向，全面贯彻“三个代表”重要思想，紧密团结在以胡锦涛为总书记的党中央周围，在中共宜春市委的领导下，紧紧依靠和团结全市人民，进一步解放思想，坚持与时俱进，以更先进的发展理念、更果敢的开放胆识、更有效的改革措施、更扎实的工作作风，创造性地做好今年政府各项工作，为全面建设小康宜春打下扎实基础，为创造宜春的美好明天而努力奋斗！
